# Consensus-building to improve implementation of NICE guidance on planning for end-of-life treatment and care: a mixed-methods study

**DOI:** 10.1186/s12904-024-01495-3

**Published:** 2024-07-13

**Authors:** Frances M. Wu, Robert Pralat, Clare Leong, Victoria Carter, Zoë Fritz, Graham Martin

**Affiliations:** 1 RAND Europe, Eastbrook House, Shaftesbury Road, Cambridge, CB2 8BF UK; 2 The Healthcare Improvement Studies (THIS) Institute, University of Cambridge, Cambridge, UK; 3 The National Institute for Health and Care Excellence, London, UK

**Keywords:** Advance care planning, End-of-life care, Communication

## Abstract

**Background:**

Despite the availability of guidance for the provision of good end-of-life care, there are significant variations across the UK in its delivery. This study sought to identify the influences on end-of-life treatment and care planning across several areas where deficiencies in evidence-based practice have been identified, and to develop consensus among healthcare providers and users for recommendations on how to address these deficits.

**Methods:**

An online survey (106 responses), qualitative interviews (55 participants) and a consensus-building exercise (475 participants in the initial round) were undertaken. Participants included people approaching the end of life, people important to them, and health and care practitioners who help people plan for the end of life or provide end-of-life care. Recruitment was via online methods, including social media and online newsletters of relevant charities and professional organisations. Thematic analysis using the framework method was used to analyse qualitative data. Synthesis of qualitative and quantitative data led to the development of statements regarding recommendations for advancing implementation of good practice. A two-stage consensus-building exercise asked respondents first to rate these statements and then to rate and rank further sub-recommendations in three areas.

**Results:**

Results from the consensus building exercise confirmed that end-of-life care planning conversations are to be welcomed and encouraged, and that the priority should be to have the conversation (which could be initiated by a range of professionals, or people planning end-of-life care themselves), rather than to wait for an ideal time to have it. Further rounds identified specific components of a standardised record of end-of-life treatment and care preferences that should be prioritised, specific health and care staff that should be empowered through training in advanced communication, and aspects of communication most important to include in training for healthcare professionals.

**Conclusions:**

Our study has identified opportunities for action to improve end-of-life treatment and care by combining multiple stakeholder perspectives and building consensus among them: the resulting recommendations have sufficient granularity to be implemented and evaluated. They are of relevance to policy makers, those who train healthcare professionals, and those looking after patients approaching the end of life.

**Supplementary Information:**

The online version contains supplementary material available at 10.1186/s12904-024-01495-3.

## Background

The implementation of evidence-based practice is challenging across healthcare fields [1], and end-of-life care (EOLC)^1^ is no exception. In the United Kingdom (UK), the National Institute for Health and Care Excellence (NICE) has produced a range of guidance and quality standards on EOLC provision. This includes guideline NG142 which focuses on the organisation and delivery of services to provide treatment and care for adults in the final weeks and months of life across all stages of care, including those relating specifically to planning ahead for EOLC which we identified as particularly important: (1) identifying people who may be approaching end of life; (2) undertaking important conversations around people’s EOLC preferences; (3) advance care planning; and (4) ensuring that preferences are documented and acted upon [2]. For this work, we employ a broad definition of ‘approaching end of life’ guided by the General Medical Council’s definition of people ‘likely to die within the next 12 months’, and including those other conditions such as advanced, progressive conditions or frailty and co-existing conditions [3].

The existing literature covers a wide range of influences across the four stages identified above. Specific characteristics of the individuals who are approaching end of life, such as the nature and course of their primary health condition, co-occurring conditions, and cultural perspectives on end of life, impact access to and uptake of EOLC in general and hospice care in particular [4–6]. Characteristics of healthcare staff – such as training and background, their own attitudes and beliefs, professional roles and how they fit into the overall pictures of an individual’s care – also contribute to timeliness of access to and decision-making surrounding EOLC [4, 6]. Other factors include the influence and support of family and caregivers, the availability of financial resources, system-level factors such as the degree of continuity and coordination of care, and broader legal and policy factors [4, 6]. 

In part due to these influences, available evidence suggests marked variations in quality of treatment and care planning at the end of life in England and the wider UK [7–11], often deviating from national standards and recommendations issued by NICE and other authorities. The Priorities for Care of the Dying Person make clear that there should be an individualised plan of care when it is thought that a person may die within the next few days or hours [12], yet the National Audit of Care at the End of Life for England and Wales (NACEL) found a documented individualised care plan in only 73% of imminent deaths [13]. In relation to communication surrounding care plans – that is, to ‘discuss, develop and review’ care plans with patients per NICE Quality Standard 144 – the 2021 NACEL found that patients were involved in care plan discussions in only 25% of all deaths. In continued efforts to address such gaps, the National Palliative and End-of-life Care Partnership has recently refreshed their framework for improving EOLC planning throughout England [14]. In Scotland, a strategy steering group led by the National Clinical Lead for Palliative Care will oversee the development and delivery of the next Palliative and End of Life Care Strategy [15], following on from the 2015 strategic framework [16].

A key first step in closing the gap between evidence-based standards and routine practice is characterising the influences on practitioners in following guidance in their day-to-day work. The overall aims of this study were twofold: to identify influences on end-of-life treatment and care planning where deficiencies in evidence-based practice have been identified; and to develop consensus from stakeholders on recommendations for approaches that might effectively address these deficits.

## Methods

Study activities included qualitative interviews, a survey, and a consensus-building exercise, described below. The research was guided throughout by two advisory groups involving patient, public, practitioner and policy stakeholders. The patient and public advisory group included eight members with a diverse range of experiences and backgrounds, including planning EOLC for themselves or for someone close. The professional advisory group comprised eight individuals from organisations with roles in end-of-life care policy and implementation, clinicians with experience in end-of-life care and care planning, and academics with expertise in the topic area.

Data collection tools were informed by early conversations with NICE, a scoping review of the literature and stakeholder interviews, which identified and verified topic areas of enquiry. Questionnaires and interview guides were drafted iteratively by the research team. Both advisory groups provided input on all the data collection tools. ZF/CL/GM/FW led interviews (two are physicians with clinical backgrounds, and two are doctorally-qualified social scientific researchers). No relationship with participants was established prior to study commencement and participants did not have knowledge of the interviewer prior to the interview.

### Initial stakeholder interviews

To ensure broad-based understanding of relevant issues, we undertook interviews with key senior stakeholders including those involved in delivering EOLC in health and social care, those representing voluntary sector organisations, and academics. Using purposive sampling, we identified relevant stakeholders in consultation with NICE and our advisory groups. All individuals were emailed to request participation in a 30–40 min online interview. Following informed consent, interviews took place and were digitally recorded and transcribed by a third-party transcription service.

### Survey and further in-depth interviews

We sought the views of a wider range of individuals involved in planning EOLC through an online survey and qualitative interviews (see Supplementary File 1 for the questionnaire and interview guide). These included: people who may be approaching the end of life or have planned ahead for this time; people important to them such as family, friends and informal carers; healthcare staff; and social care staff. For the first two groups, we sought to recruit people 18 years and older who had had conversations about EOLC in the UK within the last two years. For the latter two, we sought people who were involved in the care of people who may be approaching the end of life or receiving EOLC in the UK.

We enlisted the help of professional associations, charities and other groups with interest or involvement in EOLC to recruit participants from across the UK. Of the 75 organisations we contacted to request assistance with recruitment, 34 helped to publicise the study through social media, newsletters, bulletins, or email distribution lists. The study was also publicised on Twitter.

We administered the survey on the online Thiscovery platform (www.thiscovery.org) from December 2021 to March 2022. Survey topics included: identifying those approaching the end of life, initiating and having conversations about treatment and care preferences, documenting preferences, and ensuring that people’s preferences are known and acted on across the health and care systems. Slightly different versions of the questionnaire were used for each participant group. Survey respondents were asked if they would be interested in participating in a follow-up interview. Those who agreed were provided with further information and, subject to consent, participated in semi-structured online interviews that covered similar topics to the survey.

All interviews were digitally recorded and transcribed.

### Consensus-building exercise

We drew on the data collected in earlier stages to undertake a consensus-building exercise, using an adapted Delphi approach [17, 18], to identify the level of agreement regarding the desirability and feasibility of various approaches to improve implementation of guidance on planning EOLC. The Delphi approach is a structured methodology which seeks to build consensus by collating initial views and then allowing participants to reconsider their views in light of the wider group’s.

**Fig. 1:**
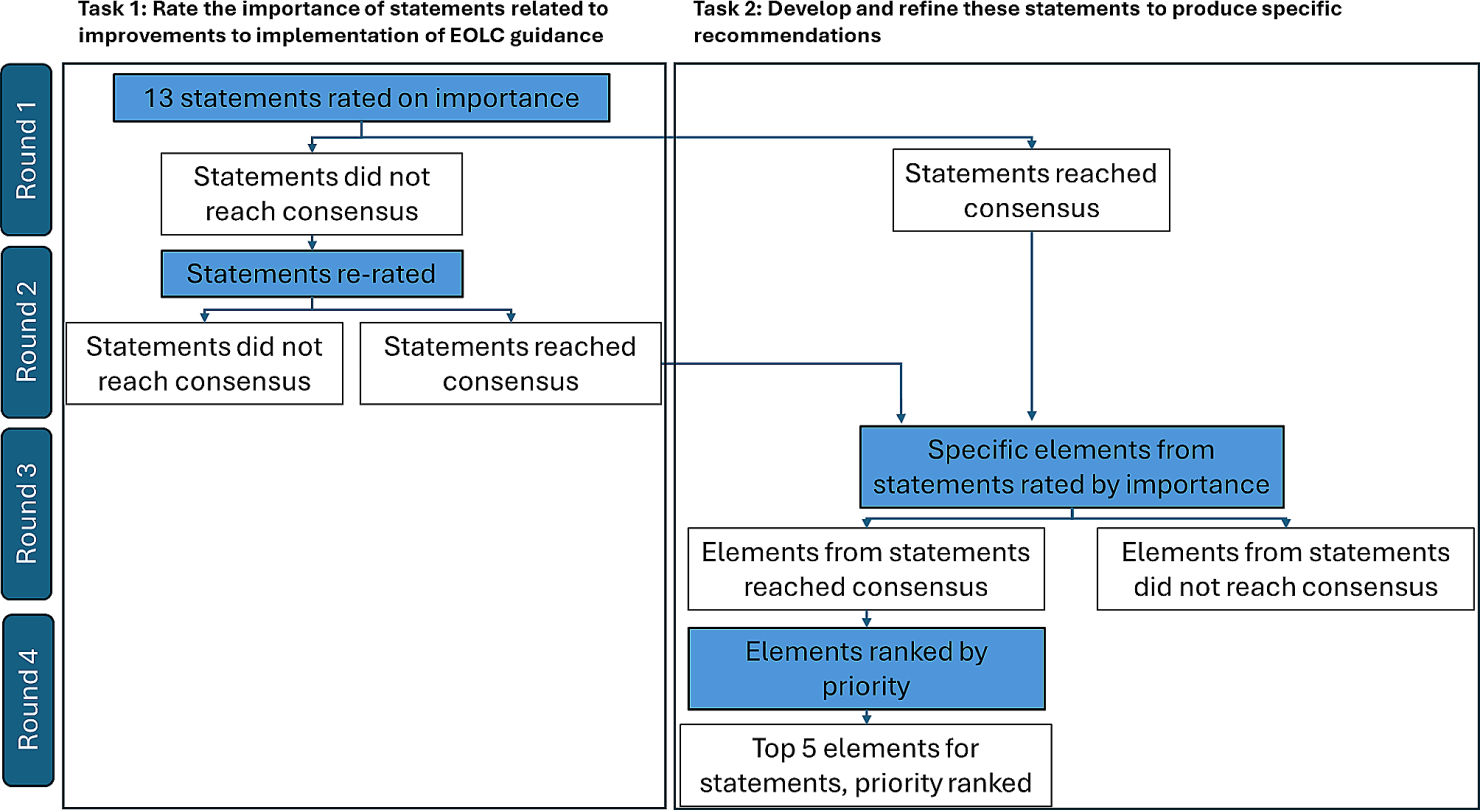
Two consensus-building tasks over four rounds, using an adapted Delphi approach

Individuals who had participated in earlier stages were invited to take part. New participants were also recruited through further publicity activities from professional associations, charities and other groups, and additional individuals representing policy or regulatory organisations with relevance to end-of-life care provision were also invited. Using the Thiscovery platform, there were two tasks over four rounds (see Fig. 1). The first task (rounds 1 and 2) was to rate the importance of a series of statements related to improvements to implementation of EOLC guidance. These statements were developed by the research team (with input from the advisory groups) based on the survey responses and the views expressed in interviews: they represented aspects of EOLC planning that were amenable to actionable improvement. The second task (rounds 3 and 4) developed and refined these statements in order to produce recommendations with sufficient specificity to be useful.

For the first round, participants were asked to rate 13 statements using a nine-point scale (1 – not important at all to 9 – extremely important). For round 2, participants were given the opportunity to revisit and re-rate the initial preferences in light of overall participant preferences - for each statement that did not meet consensus, participants were presented with their own original rating, the overall mean rating for all participants, and the mean rating for participants in their group. They were then asked to re-rate each statement in light of this information.

For round 3, participants were asked to select the top five priorities relating to specific statements and, for round 4, participants were asked to rank them.

The threshold for rating statements consensus was set at 70%, which meant that any statement where at least 70% of the respondents rated either 7, 8, 9 or 1, 2, 3 was deemed to have reached consensus that an item was, or was not, important, respectively.

### Analysis

For the survey, basic descriptive statistics (mean and proportions) were calculated for demographic questions and questions related to various aspects of end-of-life conversations.

For the qualitative interviews, a coding framework was developed using the primary areas of enquiry from the interview topic guide. Two members of the research team used the coding framework to independently code three interviews. After comparing coding, codes were slightly modified and additional codes were added to the coding framework. The remaining interviews were coded by one member of the research team. NVivo 12 was used to code the interviews and generate a matrix for framework analysis [19]. Important themes were then drawn out from analysis of the matrix.

For the consensus-building, the mean ratings were calculated for all participants by respondent group. For questions that asked participants to select their top 5 statements, we calculated for each statement the total number of participants who had included that statement among their top 5 choices. Finally for questions that asked participants to rank statements (between 1 and 5) we assigned points to the statement based on rank – 5 points for rank 1, 4 points for rank 2, through to 1 point for rank 5 – such that higher points reflect higher ranking. We calculated the total points for each statement. Analyses were performed in R and Microsoft Excel.

## Results

### Survey

We received 106 responses to the survey: 52 responses from individuals approaching the end of life or someone important to them, and 54 from health or social care staff. Demographic data are shown in Table 1.

**Table 1:** Demographic information and professional information for survey respondents, by group

Characteristic	Individuals approaching end of life and those important to them (*n* = 52)	Health/social care professionals (*n* = 54)
**Sex**		
Female	38 (73%)	45 (83%)
Male	13 (25%)	9 (17%)
Prefer not to say	1 (1%)	0 (0%)
**Age**		
18–35	4 (8%)	7 (13%)
36–45	4 (8%)	10 (19%)
46–55	7 (13%)	19 (35%)
56–65	15 (29%)	10 (19%)
66–75	12 (23%)	1 (2%)
76–85	7 (13%)	0 (0%)
86–95	1 (2%)	0 (0%)
Prefer not to say	2 (4%)	7 (13%)
**Ethnicity**		
English / Northern Irish / Scottish / Welsh / British	48 (92%)	43 (80%)
Irish	0 (0%)	1 (2%)
Any other white background	2 (4%)	3 (6%)
White and Asian	1 (2%)	2 (4%)
Indian	0 (0%)	2 (4%)
Chinese	0 (0%)	1 (2%)
Any other ethnic group	0 (0%)	1 (2%)
Prefer not to say	1 (2%)	2 (4%)
**Reported area of work for health and social care respondents (** *n* ** = 54)**		
Social care		5 (9%)
Primary care or general practice		2 (4%)
Community healthcare		7 (13%)
Acute care		22 (41%)
Secondary mental healthcare		1 (2%)
Hospice		6 (11%)
Charity sector		2 (4%)
Other		8 (16%)
No response		1 (2%)
**Reported professional role for health and social care respondents (** *n* ** = 54)**		
Doctor – consultant or GP		13 (24%)
Doctor – junior or in training		4 (7%)
Nurse		19 (35%)
Allied health professional		1 (2%)
Social worker		4 (7%)
Social care worker		2 (4%)
Advanced nurse practitioner		3 (6%)
Registered manager		3 [6]
Other		5 [9]

The 52 responses from people approaching the end of life or people important to them indicated that the individuals for whom they were responding had a range of conditions, including cancer (15, 29%), a long-term life-limiting physical condition other than cancer (24, 46%), dementia (4, 8%), a mental health condition (1, 2%) and another form of disability (3, 6%). Thirty of the 52 were people important to the person at the end of life, including family, friends or informal carers; just over half (16, 53%) of them had been assigned lasting power of attorney for health and welfare.

Around half of participants agreed or strongly agreed that they felt prepared for the conversation about EOLC when it started and that the people they spoke to were sensitive and caring. One third of participants agreed or strongly agreed they had a good understanding of services available to them and 35% agreed or strongly agreed that they felt confident that their preferences would be followed by clinicians and others providing care.

In terms of who is best placed to initiate a conversation, over half of participants felt that a member of a palliative care team specialised in EOLC, the person’s GP, someone important to the person approaching the end of life, or a member of staff at a hospital that the person has regular appointments with would all be appropriate.

Participants were asked to rate the importance of discussing and documenting various aspects of EOLC with health or social care staff. Based on percent selecting ‘Very important’, participants felt that their perspective on quality of life versus prolonging of life was highly important (85%), as were desired outcomes (85%) and specific treatments they would or would not like to receive (81%) (see Table 2). Relatively, a lower proportion of respondents rated discussing who should be present at death (56%) and preferred place of death (65%) as very important.

**Table 2:** Selected survey responses, by group

	Individuals approaching end of life and those important to them (*n* = 52)	Health/social care professionals (*n* = 54)
**Who do you think should initiate an EOLC conversation? (Percent of respondents selecting option)**		
Anyone over the age of 18	27	30
The person approaching the end of life	48	69
Someone important to the person approaching the end of life	54	72
The person’s GP	60	78
Another member of the general practice or community healthcare team	44	76
A member of social care staff, such as a social worker	27	61
A member of staff at a hospital that the person has regular appointments with	50	74
A member of staff at a hospital that the person may not know so well	21	46
A member of a palliative care team	65	70
**When do you think is the best time to have a conversation about EOLC? (Percent of respondents selecting option)**		
When it is thought that someone will die within the next year	13	20
When it is thought that someone will die within the next few days/weeks	2	0
After someone is diagnosed with a life limiting condition	44	33
Routinely with all adults	25	24
Only when initiated by the person	2	2
Other	10	20
**How important are discussing and documenting various aspects of EOLC with health or social care staff? (Percent responding Very Important)**		
Putting in place an advance care plan	75	69
People present at death	56	61
Preferred place of death	65	74
Prolonging life versus maximising quality of life	85	80
Important outcomes	85	89
Preferred treatments	81	72
Legally binding arrangements	71	44

#### Health and social care professionals

Health and social care professionals were asked similar questions to those asked of the first group, allowing comparison of some responses between groups. They recognised that several groups could be well-placed to initiate EOLC conversations – compared to people planning for EOLC and those important to them, the majority of health/social care professionals additionally indicated that another member of the general practice or community healthcare team, a member of social care staff, or the person approaching the end of life themselves would be appropriate (see Table 2). Just under half of respondents also selected a member of staff at a hospital that the person may not know so well as appropriate (see Table 2). Overall, health and social care professionals had more positive views about the appropriateness of most of the identified groups in initiating conversations, compared to people planning for EOLC and those important to them.

With regard to the content of conversations, nearly 90% of health and social care professionals rated ‘important outcomes’, and 80% rated ‘people’s views on the balance between prolonging life versus maximising quality of Iife’, as very important to discuss. Identifying who should be present at death was rated as very important by 61% of respondents, though discussing or putting in place legally binding arrangements appeared to be relatively less important for this group than for people planning EOLC and those important to them.

In terms of their own ability to discuss EOLC preferences with patients, over half of respondents strongly agreed with each of the statements that they had the right skills to start conversations and that they felt comfortable having those discussions. Only about a third of respondents strongly agreed with statements that they had access to the right tools and resources to have productive conversations and that they were confident in making treatment decisions when a patient has lost capacity.

When asked in an open-ended question what would be most helpful in identifying people approaching the end of life, having conversations about their EOLC preferences with them, or recording and sharing these preferences, one third of respondents mentioned having a nationally shared record across care services. In addition, several participants mentioned communication training, for example advanced communication to navigate difficult conversations and starting conversations with people not immediately at the end of their life.

### Interviews

We conducted 21 initial stakeholder interviews with individuals from various backgrounds, including those with experience in palliative care practice and discussing plans for EOLC, and people from voluntary and campaigning organisations with an interest in this area. Additionally, we conducted 34 interviews with individuals who responded to the survey. Of the 34 interviewees, 14 were health or social care providers, 7 were individuals planning EOLC, and 13 were carers or people important to those at the end of life. The interviews provided rich detail of people’s experience with EOLC. Below we describe findings across the interviews relating to cultural, legal and educational issues, and challenges relating to service provision and resourcing constraints. We also discuss influences that support good EOLC planning communication and approaches that healthcare professionals said they found helpful in initiating discussions about EOLC planning.

#### Cultural, legal and educational barriers to implementation of good-practice standards

Interviewees shared their views on overall challenges in terms of ensuring that people’s preferences are known, shared, and acted on. People described their sense of how the palliative care approach was often at odds with a *‘culture of healthcare to cure’*, to ‘*intervene to save lives’*.

*‘If you can’t cure, it’s to control, and then [palliative care] feels like a failure, and that’s a mindset shift. You need to be able to hold in your head success being something other than cure or control.’ (Clinical academic in palliative medicine)*.

These contrasting mindsets could, in some participants’ views, deter both healthcare professionals and patients from opening conversations about EOLC options, and result in planning beginning later than optimal. The notion that it was countercultural to integrate enabling a good experience of death within good care was expressed across participants groups. One participant, for example, described the challenge of culture change in contexts such as emergency care where clinicians are tasked with assessing a situation very rapidly and making a decision in haste:

*‘We’re in a way fighting against our historic traditional foundations of a lifesaving organisation. We’re trying to create a culture change.’ (Ambulance healthcare professional)*.

Relatedly, interviewees elaborated on how the culture of medicine was at times at odds with patient preferences. For example, some interviewees described situations where a person’s capacity was questioned simply because they did not agree with their clinicians, or chose to refuse treatment. These occurrences were, according to participants, not uncommon, despite clear legislation in England and Wales – the Mental Capacity Act – that deems that a person must be assumed to have capacity unless otherwise established. Another interviewee shared:

*‘So, I’m very, very open with doctors about talking about [my advance care plan] and they didn’t like that, he said, “Oh no, no, no, let’s not talk about that now, let’s not discuss that”. My nurse really struggled with it at first, she was like, “Well, you know, we don’t need to talk about that”,’ and then when she became more aware of my story, she’s like, “OK I understand your thinking”. But I just find that I’m more open to talking about it than most healthcare professionals know how to deal with.’ (Individual planning EOLC)*.

Several interviewees brought up areas of confusion that made difficult EOLC situations even more challenging – for those planning EOLC and professionals alike. These included which advance planning documents are legally binding and which are not, as well as the status of documented preferences regarding attempted cardio-pulmonary resuscitation (CPR) as medical recommendations rather than legally binding documents (unless expressed in an Advance Decision). Participants also described lack of understanding surrounding the legal situation when a person loses capacity about the rights (or lack thereof) of family members, particularly in instances when the wishes of family members are not aligned with healthcare professionals seeking to act in the person’s best interest, or where there are differences of opinion regarding what the patient her/himself would have wanted.

*‘[There is a] huge disjuncture between the law and medicine, how little doctors understand the law, how frequently doctors end up in court giving evidence and being cross-examined, and displaying their total ignorance about some of what are supposed to be the fundamentals of law in this country – to do with taking into account the person’s own values, wishes, feelings and beliefs, to do with the importance attached to autonomy, not simply to sanctity of life, and to do with basics like no, family are not the decision maker, the person giving the treatment is the decision maker.’ (Policy stakeholder)*.

#### Service provision and resourcing constraints on implementation of good-practice standards

Related to service provision, a particular challenge from professionals’ perspective was the tension between eliciting patient preferences and the reality of the availability of resources to meet people’s needs, and how conversations need to be bounded by system capacity (and communicated clearly) – particularly in less well-resourced areas.

*‘I think one of the initial challenges was, historically, planning for end-of-life and palliative care has been focussed around cancer and there has been inequity with non-cancer conditions. So, services would have been developed in that way and commissioned from that perspective …. [Palliative care] covers all of the different service areas, so it’s part of a care pathway for all of those conditions. So, it’s taken quite a number of years for people to recognise that it’s part and parcel of many care pathways from a service provision perspective.*’ *(Manager in palliative care)*.

#### Influences on implementing helpful EOLC planning conversations

Influences identified as facilitating good conversations in practice included: sensitivity to what is important to someone; prioritising relationship-building; ensuring individuals feel in control; demonstrating empathy and compassion; and giving people the time they need (even when time is pressing). Others included the importance of courage in communicating difficult but important news, and the need to convey positive things that could arise from good planning without sugar-coating the prognosis.

*‘You have to be prepared to say, “Going into hospital you might get better, but actually it’s a possibility you may not ever be well enough to come out of hospital and could die there”.’ (Healthcare professional in palliative medicine)*.

*‘They [hospice workers] were so good, they provided me with the suction machine, they showed me how to use it, and they said, “This is how you use it, this is when you use it, but you know that it’s not going to stop him from dying but it will make him much more comfortable when he is dying”.’ (Carer)*.

Healthcare professionals discussed approaches they had taken in initiating conversations, and some shared frameworks and specific questions asked of patients and families to gain understanding of their perspectives. Some emphasised the importance of making known how the conversation would be documented and making clear if meeting preferences may not be possible due to resources or other reasons.

Most participants agreed that advanced communication skills were critical and that observing conversations was a valuable way of learning how to facilitate conversations better. They noted, however, that a focus on the completion of some advance planning documents could impede wider communication with an individual and preclude a full understanding of what is important to them.

### Consensus-building

The consensus-building exercise took into account the main findings from the survey and interviews, specifically in areas where there would be meaningful learning or implications for EOLC. Findings from both datasets were used to identify key propositions about how practice might be improved, especially in relation to guidance about identifying people approaching the end of life, discussing with them their preferences for treatment and care, producing advance care plans, and ensuring that people’s preferences were accounted for in care provision. These were then formulated as (i) a series of statements and (ii) more detailed specifications of activities relating to some of those statements, which were considered through successive rounds of the consensus-building exercise.

#### Rounds 1 and 2 – rating of statements

There were 475 participants in the first round of consensus-building (Table 3). In the first round, 11 of the 13 statements reached consensus. Two statements (statements 5 and 7 in Table 3) did not reach consensus and were therefore subject to a second round of rating. In this second round, 60% of respondents from the first round participated (*n* = 283). Neither statement 5 nor statement 7 reached consensus, with 60.8% and 64.9% agreement respectively after the second round (see Table 3).

**Table 3:** Final overall and group-level rates of agreement for consensus building statements

	Statement	Rationale	People planning their own EOLC	People planning EOLC of others important to them	Health and social care professionals	Policymakers and representatives of organisations with interest in EOLC	All
			*n* = 278	*n* = 133	*n* = 38	*n* = 26	*n* = 475
**1**	Healthcare staff should initiate conversations and document preferences about end-of-life treatment and care planning routinely, including for people who are not yet approaching end of life – for example during regular check-ups with a GP or practice nurse, or when attending hospital appointments.	Interview data suggest that conversations about EOLC preferences often take place too late or not at all. This statement proposes that routine conversations should be occurring, suggesting that they happen regularly regardless of patients’ conditions.	76.8%	71.0%	70.3%	60.9%	73.9%
**2**	It is sometimes OK for a health or social care professional to raise the issue of planning for end-of-life treatment and care with someone, even if they don’t know the person that well.	Survey respondents showed preferences for who should initiate EOLC planning conversations, i.e. healthcare professionals that they know and see regularly. However, qualitative data suggest that conversations about EOLC preferences often take place too late or not at all. If endorsed, this statement may have value in showing health or social care professionals that it is OK to raise this issue even if they don’t know them that well.	79.4%	77.1%	78.4%	87.0%	79.1%
**3**	It is better for a health or social care professional to raise the issue of end-of-life treatment and care with someone, even if it’s not quite the ideal time, than for no-one to raise it at all.	When asked whether the initial conversation happened at the right time, survey responses were mixed. This statement seeks to address initiating EOLC planning conversations given challenges with identification of patients approaching the end of life. If endorsed, it may have value in showing health or social care professionals that it is OK to raise this issue even if they have some doubts.	84.5%	84.6%	78.4%	82.6%	83.9%
**4**	We need to empower a wider range of people, including staff, people preparing for end of life and others, to initiate conversations about end-of-life treatment and care, for example by providing them with better skills and knowledge.	Survey and interview data suggest wide ranging views on who is appropriate and qualified to initiate end-of-life care planning conversations. Among healthcare professional respondents, there was strong agreement that one thing that prevented health and social care staff from having productive conversations was the belief someone else may be best placed to have them. Yet, when asked who is appropriate to initiate these conversations, responses suggested that any healthcare professional would be appropriate. *If consensus is reached, ranking exercise in round 3 to identify/prioritise groups to be empowered.*	94.2%	92.4%	91.9%	95.7%	93.6%
**5**	Too many different guides and protocols about how to have conversations about people’s preferences around end-of-life treatment and care are available – a single guide to having conversations would be better.	Conversations planning for a person’s EOLC preferences can happen at different time points in the period before a person’s death. While the individual’s specific health condition or healthcare needs may be different, there are common elements (e.g. style, structure) of these conversations that could be included in a unified approach. *If consensus is reached, ranking exercise in round 3 to identify/prioritise elements of conversations.*	60.8% (62.1%)	60.0% (53.9%)	69.2% (56.8%)	53.3% (39.1%)	60.8% (58.2%)
**6**	Efforts to discuss and document end-of-life treatment and care preferences should focus on what matters to the individual and what they value in their life.	While survey respondents rated both preferred treatments and outcomes as very important to discuss and document, interviews with health and social care professionals suggest that EOLC planning conversations start by understanding what is most important to the person approaching the end of life. There was also some suggestion that specific directions about treatments that should and should not be given are more easily upheld if an individual loses capacity. However, we also heard from patients who found that conversations were sometimes carried out as ‘tick-box exercises’. These statements seek to see whether there is consensus on whether conversations should be values-based and/or decision-based.	94.9%	94.7%	94.6%	100.0%	95.1%
**7**	Efforts to discuss and document end-of-life treatment and care preferences should focus on specific treatment and care preferences.	Same as 6	65.5% (64.0%)	61.0% (54.2%)	61.5% (59.5%)	80.0% (60.9%)	64.9% (60.7%)
**8**	People approaching end of life are not fully aware of what cardiopulmonary resuscitation (CPR) involves or who makes the recommendation – more consistent messaging is needed.	While survey respondents rated preferred treatments as very important to discuss and document, interviews with health and social care professionals suggest that there is often too much focus on the CPR ‘decision’ during these conversations, to the neglect of wider considerations around EOLC. If endorsed, this statement may have value in showing that individuals and people who care about them should have a clear understanding of when there may be a CPR recommendation to make and when there is not.	86.2%	81.5%	86.5%	82.6%	84.8%
**9**	People approaching end of life do not have a clear understanding of what good-quality and poor-quality end-of-life treatment and care look like – more work is needed to ensure that people have clear information about what to expect at the end of life, and know where to access support when things go wrong.	Qualitative data suggested that individuals and their carers continue to have poor experiences at the end of life. Several carers spoke of difficulties providing EOLC in the home setting including access to pain medication and limited home visits. Understanding of what can be expected and potential issues with care and treatment access in various settings may need to be improved through better communication. There were a few examples where this communication occurred early and throughout the end of life period.	91.7%	86.2%	86.5%	95.7%	89.9%
**10**	Training on advanced communication skills should be provided to support healthcare professionals in initiating and conducting conversations about end-of-life treatment and care preferences.	Many survey respondents found advanced communication skills training particularly helpful to initiate sensitive or difficult conversations with patients, even given a short amount of time. They provided a very long list of resources they found helpful in this regard, making clear that there is not currently a unified approach. *If consensus is reached, ranking exercise in round 3 to identify/prioritise elements of training.*	90.6%	87.8%	78.4%	95.6%	89.1%
**11**	A single, standardised approach to documenting and recording end-of-life treatment and care preferences is needed.	Some healthcare professionals commented on how other healthcare provider notes were sometimes written in an unclear or ambiguous way. Others commented on the length of some documentation and the need for summary statements, and on important items that were not always available. Individuals and people important to them desired some feedback to confirm that their wishes had been documented or updated. *If consensus is reached, ranking exercise in round 3 to identify/prioritise what should be recorded.*	79.3%	72.9%	78.4%	73.9%	77.2%
**12**	A single integrated electronic system for recording end-of-life treatment and care preferences is needed.	Multiple respondents suggested that a record, interoperable across settings (e.g. community, hospital, ambulance), has been or would be helpful to ensure an individual’s preferences were known across the healthcare system. Qualitative data suggest that a lot of time is currently spent by some individuals to make sure patients’ wishes are known in different settings, i.e. by calling GP offices, etc. While other forms of recording preferences (e.g. ‘message in a bottle’ – a note of personal and medical information kept by individuals in the refrigerator, so that it can be easily located by ambulance staff in an emergency) are likely to be needed as a back-up, there was strong support for an integrated system, and a sense that uptake of the NHS app driven by Covid may provide an opportunity to take this forward.	82.9%	73.9%	81.1%	78.3%	80.0%
**13**	Accessing and using people’s care plans when making decisions about treatment should be routine practice in all healthcare activities.	There were concerns among some participants that consideration of preferences around EOLC was patchy among healthcare professionals when making treatment decisions. If endorsed, this statement may have value in emphasising that these preferences should be considered routinely by all healthcare professionals when making decisions about treatment when a patient lacks capacity to give consent.	94.2%	93.1%	89.2%	82.6%	93.0%

*Notes: Statements 5 and 7 underwent two rounds in the first part of consensus building. Figures shown in parenthesis reflect ratings from Round 1*

#### Rounds 3 and 4 – Priority ranking of items relating to specific statements

All 475 participants from round 1 were invited to round 3 and 57% (*n* = 273) participated. Three statements that had achieved consensus in the first task of consensus-building were covered in rounds 3 and 4 in order to develop further specificity/granularity in recommendations for these areas:

- statement 11 (*‘A single, standardised approach to documenting and recording end-of-life treatment and care preferences is needed’*).
- statement 4 (*‘We need to empower a wider range of people, including staff, people preparing for end-of-life and others, to initiate conversations about end-of-life treatment and care, for example by providing them with better skills and knowledge’*).
- statement 10 (*‘Training on advanced communication skills should be provided to support healthcare professionals in initiating and conducting conversations about end-of-life treatment and care preferences’*).

First, building on statement 11, participants were asked to rate the importance of incorporating various components into standardised documentation (using a nine-point scale, from 1 – Not important at all to 9 – Extremely important). All items reached consensus with mean ratings between 81.3% and 99.3%, including seven items with consensus above 95% (Table 3).

Second, building on statement 4, participants were asked to choose and then rank five groups of people (from a list of 11 groups) who should be prioritised in efforts to improve skills and knowledge. The top five groups selected, in order by highest to lowest rank, were: general practitioners; staff in care homes; palliative care staff; specialist nurses; and healthcare staff working in the community setting (Table 3).

Third, building on statement 10, participants were asked to choose and then rank the five most important topics (from a list of 10 topics) for advanced communication skills training. The top five topics selected, in order by highest to lowest rank, were: exploring what matters to the person and people close to them, and what concerns they might have; initiating conversations about EOLC; respecting people’s decisions about treatments they wish to receive when having conversations with them, in line with the Mental Capacity Act; facilitating and responding to questions, including signposting people to other sources of support; and talking to the person about illness progression, including prognostic uncertainty (Table 3).

## Discussion

This study identifies multiple and complex challenges in implementing good-practice recommendations in planning for end-of-life treatment, care and decision-making, particularly in the areas of initiating conversations about preferences, documenting preferences, and ensuring that people’s preferences are made known to and acted on by other health and care professionals.

Our study builds on these known challenges [4–6, 20] by identifying opportunities for action to improve end-of-life treatment and care and by combining perspectives of health and social care professionals, people planning for the end of life and those important to them, and building consensus among them to produce recommendations with sufficient granularity to be implemented and evaluated.

There was no doubt about the importance of planning for EOLC and for timely initiation of conversations across participant groups, and our study found strong consensus around the importance of raising the issue of planning for EOLC even if the perfect opportunity (in terms of time or person raising the issue) or other ideal conditions could not be found. In line with other research which suggests that patients are reluctant to initiate these conversations [21], participants agreed that health and care staff should routinely seek to open such conversations, and that a wider range of people should be empowered to initiate them. Given that many staff feel they lack the skills and confidence to have important conversations about planning EOLC [4, 22], targeted education is needed.

Further rounds of consensus-building identified and prioritised specific groups who might particularly benefit from empowerment (for example through training and development) to initiate EOLC conversations: GPs, care home staff, palliative care staff, specialty nurses as well as community-based providers. These rounds also clarified specific skills within advanced communication which were seen as particularly important to support healthcare professionals around such conversations. Finally, there was consensus that there should be a standard approach to documenting and recording preferences, and all of the components of a shared record put forward as candidate components of such records reached high consensus.

The two statements that did not reach consensus deserve brief note. Participants were not convinced that the problem of implementation was the existence of too many, competing guides and protocols about how to have conversations about EOLC options, which is interesting given the multitude and heterogeneity of guides available [23]. Nor did they agree that conversations should focus solely on specific treatment and care preferences (as opposed to broader values and preferences). This perhaps reflects the finding that while specific directives about treatment could offer a clear steer to those providing care if the individual were to lose capacity in the future, they could not readily capture all possible eventualities at a point in time, and could not in themselves do justice to the complexity of an individual’s values and preferences. Previous work has suggested that while specific ‘treatment escalation plans’ may be useful to clinicians, they are challenging to discuss with patients [24, 25]; discussions about goals of care can be more easily understood to patients [26]. 

Implications. Our findings suggest a strong appetite for undertaking conversations about planning EOLC early, and by a range of individuals who may find themselves in positions to initiate these discussions but who, our survey suggests, may not be certain that they are the best-placed person to do so. Our work offers a priority of suggested health and care staff groups who would particularly benefit from training in this area, as well as specific aspects of communication training which are critical to include and which may address issues of confidence and knowledge surrounding conversations about planning EOLC. Professional associations such as Royal Colleges might consider these suggestions in terms of the continuing professional development support they offer to their members. Educational bodies should consider integrating teaching end-of-life conversations, with the domains that we have identified, into their courses.

The desire to have a “*‘A single, standardised approach to documenting and recording end-of-life treatment and care preferences is needed”* should be heeded. The Parliamentary and Health Service Ombudsman recent report [27] suggests this, and our research provides further evidence that this is needed, alongside data on what such a document should look like. Most of the fields align with the ReSPECT process, which was recommended for use by the Care Quality Commission and Ombudsman [27, 28]; future iterations of ReSPECT should incorporate evidence from this study.

Strengths of this study include its responsive nature and the wide range of participants involved across study activities. The study also has important limitations. Participants in all stages were self-selecting – they were likely to have an interest in the EOLC and experience in talking about it, and may potentially have been more positively inclined towards the importance of discussing and planning EOLC than the wider population. The number of survey respondents was modest and interviewees were predominantly white. The representativeness of the sample is thus limited. While there were greater number of participants for consensus-building, the recruitment approach (for example, through charities with an interest in promoting understanding of and proactive engagement in EOLC issues) may mean that participants’ views are not typical of the wider population. Finally, for all study activities, participation from professionals from social care backgrounds was relatively limited.

## Conclusions

We employed a multi-method approach including a survey, interviews, and a consensus-building exercise to identify and understand key influences on implementation of EOLC guidance and build consensus on what is needed to advance good practice in EOLC planning. Results from the consensus-building exercise confirmed that EOLC planning conversations are to be welcomed and encouraged, and that the priority should be to have the conversation (which could be initiated by a range of professionals, or people planning EOLC themselves), rather than to wait for an ideal time to have it. Further rounds identified specific components of a standardised record of EOLC treatment and care preferences that should be prioritised, specific health and care staff who should be empowered through training in advanced communication, and aspects of communication most important to include in training for healthcare professionals.

### Electronic supplementary material

Below is the link to the electronic supplementary material.

Supplementary Material 1

## Data Availability

Due to the terms of consent, data from interviews are not available. Data from the survey and consensus-building work may be made available to other researchers upon reasonable request from GM.

## Footnotes

1 We use the abbreviation EOLC throughout the paper, which we mean to denote both care and treatment at the end of life.

## References

[1] Braithwaite J, Glasziou P, Westbrook J (2020). The three numbers you need to know about healthcare: the 60-30-10 challenge. BMC Med.

[2] National Institute for Health and Care Excellence. NICE guideline NG142 End of life care for adults: service delivery. 2019. 31633897

[3] General Medical Council, Guidance. Treatment and care towards the end of life 2010 [updated 15 March 2022. https://www.gmc-uk.org/professional-standards/professional-standards-for-doctors/treatment-and-care-towards-the-end-of-life/guidance.

[4] Threapleton DE, Chung RY, Wong SYS, Wong ELY, Kiang N, Chau PYK (2017). Care toward the end of life in older populations and its implementation facilitators and barriers: a scoping review. J Am Med Dir Assoc.

[5] Tobin J, Rogers A, Winterburn I, Tullie S, Kalyanasundaram A, Kuhn I, Barclay S. Hospice care access inequalities: a systematic review and narrative synthesis. BMJ Supportive &amp; Palliative Care. 2022;12(2):142. 10.1136/bmjspcare-2020-002719 PMC9125370 33608254

[6] Travers A, Taylor V (2016). What are the barriers to initiating end-of-life conversations with patients in the last year of life?. Int J Palliat Nurs.

[7] Care Quality Commission. A different ending: Address inequalities in end of life care - Overview report 2016 [ https://www.cqc.org.uk/sites/default/files/20160505%20CQC_EOLC_OVERVIEW_FINAL_3.pdf.

[8] Office of National Statistics. National Survey of Bereaved People (VOICES). England 2015 [ https://www.ons.gov.uk/peoplepopulationandcommunity/healthandsocialcare/healthcaresystem/bulletins/nationalsurveyofbereavedpeoplevoices/england2015.

[9] House of Commons Health Committee (2015). End-of-life care: Fifth Report of Session 2014-15.

[10] Parliamentary and Health Service Ombudsman. Dying without dignity. Investigations by the Parliamentary and Health Service Ombudsman into complaints about end of life care. 2015.

[11] Lasserson DS, Subbe C, Cooksley T, Holland M (2019). SAMBA18 Report - A National Audit of Acute Medical Care in the UK. Acute Med.

[12] Leadership Alliance for the Care of Dying People. One chance to get it right: Improving people’s experience of care in the last few days and hours of life. 2014.

[13] National Audit of Care at the End of Life. Third round of the audit (2021/22) report - England and Wales. NHS Benchmarking Network; 2022.

[14] National Palliative and End of Life Care Partnership. Ambitions for Palliative and End of Life Care: A national framework for local action 2021–2026. 2021.

[15] Scottish Government Healthcare Quality and Improvement Directorate. Palliative and end of life care strategy: aims, principles and priorities 2023 [ https://www.gov.scot/publications/palliative-and-end-of-life-care-strategy-aims-principles-and-priorities/.

[16] Scottish Government Population Health Directorate. Palliative and end of life care: strategic framework for action 2015 [ https://www.gov.scot/publications/strategic-framework-action-palliative-end-life-care/.

[17] Dalkey N, Helmer O (1963). An experimental application of the DELPHI Method to the Use of experts. Manage Sci.

[18] Parks S, d’Angelo C, Gunashekar S. Citizen science: generating ideas and exploring consensus. The Healthcare Improvement Studies Institute, University of Cambridge. 2018:1–16.

[19] Gale NK, Heath G, Cameron E, Rashid S, Redwood S (2013). Using the framework method for the analysis of qualitative data in multi-disciplinary health research. BMC Med Res Methodol.

[20] Lund S, Richardson A, May C (2015). Barriers to advance care planning at the end of life: an explanatory systematic review of implementation studies. PLoS ONE.

[21] Almack K, Cox K, Moghaddam N, Pollock K, Seymour J (2012). After you: conversations between patients and healthcare professionals in planning for end of life care. BMC Palliat Care.

[22] Blackwood DH, Walker D, Mythen MG, Taylor RM, Vindrola-Padros C (2019). Barriers to advance care planning with patients as perceived by nurses and other healthcare professionals: a systematic review. J Clin Nurs.

[23] Ramirez-Valdez EA, Leong C, Wu F, Ball S, Maistrello G, Martin G, Fritz Z (2022). Towards cataloguing and characterising advance care planning and end-of-life care resources. BMC Palliat Care.

[24] Chen W, Chung JOK, Lam KKW, Molassiotis A (2023). End-of-life communication strategies for healthcare professionals: a scoping review. Palliat Med.

[25] Warner BE, Lound A, Grailey K, Vindrola-Padros C, Wells M, Brett SJ (2023). Perspectives of healthcare professionals and older patients on shared decision-making for treatment escalation planning in the acute hospital setting: a systematic review and qualitative thematic synthesis. EClinicalMedicine.

[26] Larson DG, Tobin DR (2000). End-of-life conversations: evolving practice and theory. JAMA.

[27] Parliamentary and Health Service Ombudsman. End of life care: improving DNACPR conversations for everyone [ https://www.ombudsman.org.uk/sites/default/files/End_of_life_care_improving_do_not_attempt_CPR_conversations_for_everyone.pdf.

[28] Care Quality Commission. Protect, respect, connect - decisions about living and dying well during COVID-19 2021 [ https://www.cqc.org.uk/publications/themed-work/protect-respect-connect-decisions-about-living-dying-well-during-covid-19.

