# Supplementary material for: Consensus-building to improve implementation of NICE guidance on planning for end-of-life treatment and care: a mixed-methods study
Source: BMC Palliat Care. 2024 Jul 13;23:169. doi: 10.1186/s12904-024-01495-3 (PMC11245782; doi:10.1186/s12904-024-01495-3)
Supplement: Supplementary file 1 — Supplementary Material 1 [file 12904_2024_1495_MOESM1_ESM.docx]

## Appendix 1 – Study questionnaires

End-of-life care planning

Questionnaire (groups ii and iii – people planning for

end-of-life care and those important to them)

Version 1.0

##### Have you had a conversation with health or social care staff about planning end-of- life treatment and care?

Response options (multiple choice – can select one only):

- 1. Yes – my own end-of-life treatment and care
  2. Yes – the end-of-life treatment and care of someone important to me
  3. Yes – both my own end-of-life treatment and care and that of someone important to me
  4. No

*Participants who respond with options a or c proceed to question 2a. Participants who respond with option b are routed to question 2b.*

*Participants who respond ‘No’ are routed to question 3.*

##### 2a. To what extent do you agree or disagree with each of the following statements about the conversations you have had with health or social care staff about planning for your end-of-life treatment and care?

##### If you have had more than one conversations about your own care, please answer the questions while considering the first time you had a conversation like this.

- I felt prepared for the conversation when it started.
- I was able to control what we talked about during the conversation.
- We discussed several possibilities for treatment and care as I approach the end of my life.
- At the end of the conversation, I felt that I had a good understanding of different services available to help with my end-of-life treatment and care.
- The conversation covered the things that mattered to me most about my end-of-life treatment and care.
- The conversation happened at about the right time.
- The person or people I spoke to was sensitive and caring during the conversation.
- At the end of the conversation, I felt that I had a good understanding of what needed to happen next to ensure that my end-of-life care preferences would be met.
- I feel confident that, as far as possible, my preferences will be followed as I approach the end of life.

For each question: five-point Likert scale (Strongly disagree, Disagree, Neither agree nor disagree, Agree, Strongly agree) plus ‘Don’t know’ option. Optional free text box for further comment.

*Participants are then routed to question 3.*

##### 2b. To what extent do you agree or disagree with each of the following statements about the conversations you have had with health or social care staff about planning end-of-life treatment and care for someone important to you?

##### If you have had more than one conversations about your own care, please answer the questions while considering the first time you had a conversation like this.

- I felt prepared for the conversation when it started.
- The person important to me and I were able to control what we talked about during the conversation.
- We discussed several possibilities for treatment and care of the person important to me as they approach the end of their life.
- At the end of the conversation, I felt that I had a good understanding of different services available to help with the end-of-life treatment and care of the person important to me.
- The conversation covered the things that mattered most about end-of-life treatment and care to the person important to me.
- The conversation happened at about the right time.
- The person I spoke to was sensitive and caring during the conversation.
- At the end of the conversation, I felt that I had a good understanding of what needed to happen next to ensure that the end-of-life care preferences of the person important to me would be met.
- I feel confident that, as far as possible, the preferences of the person important to me will be followed as they approach the end of life.

For each question: five-point Likert scale (Strongly disagree, Disagree, Neither agree nor disagree, Agree, Strongly agree) plus ‘Don’t know’ option. Optional free text box for further comment.

##### When do you think is the best time to have an initial conversation with a member of health or social care staff about end-of-life care preferences?

Response options (multiple choice – can select only one):

- - Routinely with all adults, regardless of current age, health, or medical conditions
  - Soon after someone is diagnosed with a condition that may shorten their life, or other factors are present that might increase their risk of dying
  - When it is thought that someone might die within the next 6-12 months
  - When it is thought that someone might die within the next 1-6 months
  - When it is thought that someone might die within the next few days to weeks
  - Only when initiated by the person themselves, regardless of their stage of life
  - I do not think that people should have conversations about end-of-life care preferences
  - Don’t know
  - Other (with free text box)

##### Who do you think should initiate a conversation about end of life treatment and care preferences?

Response options (multiple choice – can select more than one):

- - The person approaching the end of their life
  - Someone important to the person approaching the end of their life, such as a family member or close friend
  - The person’s general practitioner
  - Another member of the general practice or community healthcare team, such as a practice nurse or district nurse
  - A member of social care staff, such as a social worker
  - A member of staff at a hospital that the person has regular appointments with *(for example, for planned visits to the outpatients clinic)*
  - A member of staff at a hospital that the person may not know so well *(for example, for unplanned care in the emergency department)*
  - A member of a palliative care team, specialised in end-of-life care
  - Don’t know
  - Someone else (with free text box) Free text box for further comment

*Participants who responded to question 1 with options a, c or d proceed to question 5a. Participants who responded to question 1 with option b are routed to question 5b.*

##### 5a. How important is it to you to discuss and document each of the following aspects of end-of-life treatment and care with a member of health or social care staff?

- - Who should be present with me at the time of my death
  - My preferred place of death
  - My views on the balance between prolonging life as much as possible, versus maximising quality of life
  - Which outcomes are most important to me – for example my comfort, or my day-to-day independence
  - What specific treatments I would or wouldn’t like to receive – for example ventilation to help me stay alive, or cardiopulmonary resuscitation to try to revive me if my heart stops or I stop breathing

For each question: four-point Likert scale (Unimportant, Not very important, Important, Very important) plus ‘Don’t know’ option. Free text box to add other things not listed.

*Participants are then routed to question 6.*

##### 5b. How important is it to you that each of the following aspects of end-of-life treatment and care are discussed and documented for the person important to you with a member of health or social care staff?

- - Who should be present with them at the time of their death
  - Their preferences about relief from pain and other symptoms
  - Their preferred place of death
  - Their views on the balance between prolonging life as much as possible, versus maximising quality of life
  - Which outcomes are most important to them – for example their comfort, or their day- to-day independence
  - What specific treatments they would or wouldn’t like to receive – for example ventilation to help them stay alive, or cardiopulmonary resuscitation to try to revive them if their heart stops or they stop breathing

For each question: four-point Likert scale (Unimportant, Not very important, Important, Very important) plus ‘Don’t know’ option. Free text box to add other things not listed.

##### Are there any approaches or resources for discussing and planning end-of-life care for yourself or someone important to you that you have found particularly useful? Please give details here.

Free-text box

##### What would be most helpful to you in planning for end-of-life treatment and care for yourself or someone important to you?

Free-text box

##### Have you or someone important to you used the Recommended Summary Plan for Emergency Care and Treatment (ReSPECT) process to help in planning end-of-life care? If so, would you be happy to answer some extra questions about this?

*To include an image of the ReSPECT form as an aide-memoire for participants.*

Response options (multiple choice – can only select one):

- - I haven’t used ReSPECT
  - I have used ReSPECT and would be happy to answer some questions about it
  - I have used ReSPECT but would prefer not to answer questions about it

*Participants who select ‘I haven’t used ReSPECT’ or ‘I have used ReSPECT but would prefer not to answer questions about it’ are routed to question 16.*

*Participants who select ‘I have used ReSPECT and would be happy to answer some questions about it’ proceed to question 9.*

##### We are interested in knowing more about your experience with ReSPECT.

##### Please can you tell us when you had your ReSPECT conversation?

- - During an emergency admission to hospital
  - When I or the person important to me was given a new diagnosis in hospital, or in an outpatient setting
  - At my GP practice (or the GP practice of the person important to me) while attending specifically to plan for future treatment and care
  - At my GP practice (or the GP practice of the person important to me) while attending for another reason
  - Other (free-text box)

##### With whom did you have the ReSPECT conversation?

- - With my GP (or the GP of the person important to me)
  - With a hospital doctor
  - With a specialist nurse
  - With someone else (free-text box)

##### How satisfied were you with the ReSPECT conversation overall?

- - Very satisfied
  - Quite satisfied
  - Neither satisfied nor dissatisfied
  - Quite dissatisfied
  - Very dissatisfied

##### Please add any comments on your response here:

Free-text box.

##### Did you feel you had the opportunity to express your views during the ReSPECT conversation?

- - Very much so
  - Somewhat so
  - A little
  - Not at all
  - Not sure

##### Please add any comments on your response here:

Free-text box.

##### Did you feel that the recommendations recorded in the ReSPECT form reflected your wishes, or the wishes of the person important to you?

- - Very much so
  - Somewhat so
  - A little
  - Not at all
  - Not sure

##### What, if anything, do you think is good about the ReSPECT process?

Free-text box.

##### What, if anything, could be done to improve the ReSPECT process?

Free-text box.

##### We are almost at the end of the questionnaire. Is there anything else you would like to comment relating to planning end-of-life treatment and care preferences?

Free text box

##### Finally, we would like to ask some questions about you and your background. You do not have to answer any of these questions.

##### What is your sex?

Response options (multiple choice – can select only one):

- - Female
  - Male
  - Prefer not to say

##### What is your age?

Response options (multiple choice – can select only one):

- - 18-35
  - 36-45
  - 46-55
  - 56-65
  - 66-75
  - 56-85
  - 86-95
  - 96 or over
  - Prefer not to say

##### What is your ethnic group?

Response options (multiple choice – can select only one):

- - White – English / Northern Irish / Scottish / Welsh / British
  - White – Irish
  - White – Gypsy or Irish Traveller
  - White – Any other white background
  - Mixed/multiple ethnic groups – White and Black Caribbean
  - Mixed/multiple ethnic groups – White and Black African
  - Mixed/multiple ethnic groups – White and Asian
  - Mixed/multiple ethnic groups – Any other mixed background
  - Asian/Asian British – Indian
  - Asian/Asian British – Pakistani
  - Asian/Asian British – Bangladeshi
  - Asian/Asian British – Chinese
  - Asian/Asian British – Any other Asian background
  - Black / African / Caribbean / Black British – African
  - Black / African / Caribbean / Black British – Caribbean
  - Black / African / Caribbean / Black British – Any other Black/African/Caribbean background
  - Other ethnic group – Arab
  - Other ethnic group – Any other ethnic group
  - Prefer not to say

##### Previous research has suggested that some groups of people may be at risk of unequal access to end-of-life care. We would like therefore like to collect some information about you and your background. Which, if any, of the following statements apply to you?

Response options (multiple choice – can select more than one):

- - I have cancer
  - I have a long-term life-limiting physical condition other than cancer
  - I have dementia
  - I am lesbian, gay or bisexual
  - I am transgender
  - I have a learning disability
  - I have a mental health condition
  - I am or have been homeless
  - I live or have lived in a secure or detained setting, such as a prison or detention centre
  - None of these

End-of-life care planning

Questionnaire (groups iv and v – health and social care staff)

Version 1.0

##### Please indicate which of these statements most accurately describes your role in helping people with their preferences for end-of-life treatment and care.

Response options (multiple choice – can select one only):

- 1. I am involved in helping people to plan for their end-of-life treatment and care, but not in delivering it.
  2. I am involved in delivering treatment and care to people towards the end of their lives, but not in helping people plan for this process.
  3. I am involved in both helping to plan end-of-life treatment and care, and delivering it.
  4. I am not involved in planning or delivering end-of-life care and treatment.

*Participants who select options a and c proceed to question 2. Participants who select options b and d are routed to question 8.*

1. Questions for participants with experience of initiating and having conversations with people nearing the end of life

##### Have you been involved in a conversation with someone about planning their end-of- life treatment and care in the last two years?

Response options (multiple choice – can select one only):

- Yes
- No

##### To what extent do you agree with the following statements about identifying people nearing the end of life and initiating conversations with them about their end-of-life treatment and care preferences?

- I am confident that I can correctly identify people who may be entering the last year of life.
- I feel confident in my ability to initiate conversations with people approaching end of life about their treatment and care preferences.
- For at least some people, I am the right person to initiate a conversation about end-of- life treatment and care preferences.
- I feel I know what is most important to discuss with people approaching end of life.

For each question: five-point Likert scale (Strongly disagree, Disagree, Neither agree nor disagree, Agree, Strongly agree) plus ‘Don’t know’ option. Optional free text box for further comment.

##### Are there any approaches or resources for trying to i dentify people who might be approaching the last year or months of their life that you have found particularly useful? Please give details here.

(Free text box)

##### Are there any approaches or resources for d iscussing and planning end-of-life treatment and care with patients or service users that you have found particularly useful? Please give details here.

(Free text box)

##### Are there any approaches or resources that you have found particularly useful for ensuring that people’s preferences for end-of-life treatment and care are r ecorded,

##### s hared and used by others? Please give details here.

(Free text box)

##### What would be most helpful to you in i dentifying people approaching end of life,

##### h aving conversations about their end-of-life treatment and care preferences with them, and r ecording and sharing these preferences?

(Free text box)

1. General questions on challenges in planning end-of- life care

##### When do you think is the best time for a health or social care professional to have an initial conversation with a patient or service user about end-of-life care preferences?

Response options (multiple choice – can select only one):

- Routinely with all adults, regardless of current age, health, or medical conditions
- Soon after someone is diagnosed with a condition that may shorten their life, or other factors are present that might increase their risk of dying
- When it is thought that someone might die within the next 6-12 months
- When it is thought that someone might die within the next 1-6 months
- When it is thought that someone might die within the next few days to weeks
- Only when initiated by the person themselves, regardless of their stage of life
- I do not think that people should have conversations about end-of-life care preferences
- Don’t know
- Other (with free text box)

##### Who do you think should initiate a conversation about end of life treatment and care preferences?

Response options (multiple choice – can select more than one):

- The person approaching the end of their life
- Someone important to the person approaching the end of their life, such as a family member or close friend
- The person’s general practitioner
- Another member of the general practice or community healthcare team, such as a practice nurse or district nurse
- A member of social care staff, such as a social worker
- A member of staff at a hospital that the person has regular appointments with (for example, during planned visits to the outpatients clinic)
- A member of staff at a hospital that the person may not know so well (for example, during unplanned care in the emergency department)
- A member of a palliative care team, specialised in end-of-life care
- Don’t know
- Someone else (with free text box)

##### To what extent do you agree or disagree with each of the following statements about discussing end-of-life treatment and care preferences with patients or service users?

- I feel I have the right skills to start conversations with people about their end-of-life treatment and care.
- I feel comfortable discussing people’s end-of-life treatment and care preferences with them.
- I have access to the right tools and resources to have productive conversations with people about their end-of-life treatment and care preferences.
- I am confident that the preferences expressed by people will be used by healthcare staff providing end-of-life treatment and care.
- I am confident that the preferences expressed by people will be used by social care staff providing end-of-life care.

For each question: five-point Likert scale (Strongly disagree, Disagree, Neither agree nor disagree, Agree, Strongly agree) plus ‘Don’t know’ option. Optional free text box for further comment.

##### To what extent do you think the following issues are important in preventing health and social care staff from having productive conversations about people’s end-of-life care preferences?

- Uncertainty over whether someone is approaching end of life
- Lack of time to have a productive conversation
- Lack of confidence in raising sensitive issues
- Lack of knowledge about what to discuss
- Lack of knowledge about the options and services available
- Uncertainty over what to do with what is discussed
- Uncertainty over whether and how the information will be acted on by others in the health and social care system
- Belief that someone else may be best placed to have conversations of this kind
- Difficulty in identifying individuals in particular groups who may be approaching end of life (free text box stating which group/s)
- Other (with free text box)

For each question: four-point Likert scale (Unimportant, Not very important, Important, Very important) plus ‘Don’t know’ option.

##### How important is it to plan ahead with a patient or service user about each of the following aspects of end-of-life treatment and care?

- Their preferences for who should be present with them at the time of their death
- Their preferred place of death
- Their views on the balance between prolonging life as much as possible, versus maximising quality of life
- Which outcomes are most important to them – for example their comfort, or their day- to-day independence
- What specific treatments they would or wouldn’t like to receive – for example ventilation or cardiopulmonary resuscitation to try to revive them
- Other

For each question: four-point Likert scale (Unimportant, Not very important, Important, Very important) plus ‘Don’t know’ option. Free text box to add other things not listed.

Free-text box

*Participants who responded to question 1 with options a and d are routed to the ReSPECT questions, starting question 17.*

*Participants who responded to question 1 with options b and c proceed to question 13.*

1. Questions on delivering end-of-life care according to people’s preferences

##### How challenging do you find the following issues when trying to ensure that people’s preferences about end-of-life treatment and care are shared with and used by other practitioners?

1. People’s preferences regarding their end-of-life treatment and care are not recorded appropriately in the first place.
2. The records we have regarding people’s end-of-life treatment and care preferences are not

routinely updated, and so may be out of date.

1. The records we have regarding people’s end-of-life treatment and care preferences are not always shared with the right people and organisations.
2. The records we have regarding people’s end-of-life treatment and care preferences are not acted upon at the right time, for example regarding admissions, place of death or access to hospice and palliative care
3. Other (free text box)

For each question: four-point Likert scale (Not at all challenging, A little bit challenging, Quite challenging, Very challenging) plus ‘Don’t know’ option. Optional free text box for further comment.

##### When caring for someone who is approaching the end of their life, how easy do you find it to access up-to-date records of their preferences?

Response options (multiple choice – can only select one):

- Very difficult
- Quite difficult
- Neither easy nor difficult
- Quite easy
- Very easy
- Not sure

Please comment on the reasons for your answer (free text box)

##### Are there any approaches or resources for a ccessing and making use of people’s preferences for treatment and care towards the end of life that you have found particularly helpful? Please give details here.

##### What would be most helpful to you in accessing and making use of people’s preferences when delivering treatment and care to people approaching end of life?

Free-text box

1. Questions on the ReSPECT process

##### Have you or someone important to you used the Recommended Summary Plan for Emergency Care and Treatment (ReSPECT) process to help in planning end-of-life care? If so, would you be happy to answer some extra questions about this?

*To include an image of the ReSPECT form as an aide-memoire for participants.*

Response options (multiple choice – can only select one):

1. I haven’t used ReSPECT
2. I have used ReSPECT and would be happy to answer some questions about it
3. I have used ReSPECT but would prefer not to answer questions about it

*Participants who select options a or c are routed to question 21. Participants who select option b proceed to question 18.*

##### To what extent do you agree with the following statements about the ReSPECT process?

- It helps me to identify people who would benefit from planning about care and treatments towards the end of life
- It helps me to conduct useful conversations with people about care and treatment towards the end of their lives
- It helps me to document recommendations about people’s preferences for care and treatment towards the end of their lives

For each question: five-point Likert scale (Strongly disagree, Disagree, Neither agree nor disagree, Agree, Strongly agree) plus ‘Don’t know’ option. Free text box for further comment alongside each of the three statements.

##### What, if anything, do you think is good about the ReSPECT process? (free text)

##### What, if anything, could be done to improve the ReSPECT process? (free text)

1. Final questions

##### Is there anything else you would like to comment relating to identifying people approaching end of life, having productive conversations about their preferences, and delivering treatment and care to them?

Free text box

##### Finally, we would like to ask some questions about you and your background. You do not have to answer any of these questions.

##### Which of the following areas do you work most in?

Response options (multiple choice – can only select one):

- Social care
- Primary care or general practice
- Community healthcare
- Acute care
- Secondary mental healthcare
- Other (with free text box)

##### Which of the following best describes your professional role?

Response options (multiple choice – can only select one):

- Doctor – consultant or GP
- Doctor – junior or in training
- Nurse
- Healthcare assistant or nursing assistant
- Allied health professional
- Social worker
- Social care worker
- Other (with free text box)

##### What is your sex?

Response options (multiple choice – can select only one):

- Female
- Male
- Prefer not to say

##### What is your ethnic group?

Response options (multiple choice – can select only one):

- White – English / Northern Irish / Scottish / Welsh / British
- White – Irish
- White – Gypsy or Irish Traveller
- White – Any other white background
- Mixed/multiple ethnic groups – White and Black Caribbean
- Mixed/multiple ethnic groups – White and Black African
- Mixed/multiple ethnic groups – White and Asian
- Mixed/multiple ethnic groups – Any other mixed background
- Asian/Asian British – Indian
- Asian/Asian British – Pakistani
- Asian/Asian British – Bangladeshi
- Asian/Asian British – Chinese
- Asian/Asian British – Any other Asian background
- Black / African / Caribbean / Black British – African
- Black / African / Caribbean / Black British – Caribbean
- Black / African / Caribbean / Black British – Any other Black/African/Caribbean background
- Other ethnic group – Arab
- Other ethnic group – Any other ethnic group
- Prefer not to say

End-of-life care planning

Topic guide (group i – individuals from stakeholder organisations)

Version 1.0

Introductory questions

We’re interested in talking to you about your knowledge and experience of planning for care and treatments at the end of life, and how people in the health and social care system support this process and ensure that people’s plans are acted on. I know this isn’t the easiest thing to talk about, so if there are any questions you don’t want to answer, that’s absolutely fine. If you need a break, just let me know. And if you want to stop the interview altogether, that’s no problem at all – you don’t need to give a reason and it won’t affect your employment.

1. Please could you tell me a little about yourself and your background, particularly as it relates to your interest in and knowledge about end-of-life care.
2. What, if anything, do you know about the guidance from the National Institute for Health and Care Excellence (NICE) about end-of-life care provision?
   1. Specifically, what do you know about NICE’s guidance on supporting people in planning for care at the end of their lives?
   2. [Reassure if necessary that lack of knowledge about NICE guidance is not an issue for participation]
3. In general terms, what do you see as the key challenges in ensuring that people’s preferences about end-of-life care are understood and respected by the healthcare system?

Questions on key activities in planning for end-of-life care

In this study, we are particularly interested in the work involved in ensuring that people are able to make effective preparations for care and treatments at the end of life. This means, for example, giving people the opportunity to discuss their preferences with care providers, ensuring that these preferences are properly recorded and shared, and making sure that they are accessed and used by health and social care staff when caring for those people. We’d like to ask you questions about the various stages involved in this process. We realise that you may not know about all of these stages: if there are some questions you don’t feel well placed to answer, that’s fine – just let me know and we’ll move on to the next area.

1. First of all, **identifying people who may be coming towards the end of their life** – for example, entering what is likely to be the final year of their life. Can you tell me what you know about
   1. how healthcare providers identify people who might be in their final year of life?
   2. How healthcare providers might ensure that individuals are assessed for their end- of-life care and treatment needs in a timely and consistent manner?
2. Do you have any direct experience of this process?
3. What do you see as the key challenges in identifying people who may be approaching end of life?
   1. What (if any) do you see as the issues with systematically screening patient notes or data to identify people approaching end of life?
   2. What (if any) do you see as the issues with identifying people opportunistically when they come into contact with the health service (for example appointments with GPs or attendance at the ED)?
   3. What (if any) do you see as the issues with relying on patients and carers to identify who is approaching end of life?
4. Are there any particular groups who you think may be less easy to identify?
   1. Which groups and why?
   2. Can you think of ways of addressing this?
5. What would you see as important in making the identification process work well, for patients, carers and staff?
6. Are you aware of any tools or resources for this process?
   1. What are they?
   2. What do you see as their strengths and weaknesses?
7. Second, I’d like to ask you about **how to go about having important conversations with**

**people who may be approaching the end of their life** about their preferences and needs for their care and treatment. Can you tell me what you know about how healthcare providers try to do this?

1. Do you have any direct experience of this process?
2. What are the important considerations in *initiating* conversations like this?
   1. What worked/has worked well in the past and what didn’t?
   2. Who do you feel is best placed to initiate these conversations – for example, a particular kind of healthcare professional, or someone with a particular relationship with the person nearing end of life?
3. What do you think are the most important things that the patient should be asked about in a conversation like this? [Prompt as necessary regarding:
   1. shared understanding of the diagnosis and prognosis
   2. goals of care and treatment –
      1. health outcomes important to the individual
      2. whether to prioritise extending life or to prioritise comfort and quality of life
   3. circumstances under which hospital admission should or should not be considered
   4. cardiopulmonary resuscitation, and any other specific treatments which the patient would or would not wish to receive
   5. preferred location of care
   6. involvement of family and carers
   7. other considerations]
4. What do you see as the main challenges in having these conversations? What do you think puts patients off having them? What do you think puts clinicians off having them?
5. What would you see as important in making these conversations go well, for patients, carers and staff?
6. Are you aware of any tools or resources for this process?
   1. What are they?
   2. What do you see as their strengths and weaknesses?
7. Next, I have some questions specifically about **advance care planning** – that is, how people would like to be cared for specifically in the event that they are no longer able to communicate their preferences. Can you tell me what you know about this process?
8. Do you have any direct experience of advance care planning?
9. What are your views on how advance care plans should be documented?
10. What, to your knowledge, are the main challenges in doing advance care planning well?
11. Have you used any tools or resources in advance care planning?
    1. What are they?
    2. What do you see as their strengths and weaknesses?
12. Last in terms of key activities in planning for end-of-life treatment and care, I’d like to ask

you about **ensuring that people’s preferences are shared with and used** by other practitioners. Can you tell me what you know about this?

1. Do you have any direct experience of this process?
2. What do you see as the main challenges in:
   1. documenting people’s preferences appropriately?
   2. ensuring they are shared with the right people and organisations?
   3. ensuring they are acted upon at the right time in people’s end-of-life care?
   4. ensuring they result in access to the right services, for example hospice and palliative care?
3. What tends to help in:
   1. documenting people’s preferences appropriately?
   2. ensuring they are shared with the right people and organisations?
   3. ensuring they are acted upon at the right time in people’s end-of-life care?
   4. ensuring they result in access to the right services, for example hospice and palliative care?
4. In your experience, what steps are in place to ensure that copies of advance care plans are kept at a person’s place of residence and are accessible upon admission to a hospital, care home or hospice?
5. Are you aware of any tools or resources to help people share and make use of end-of-life care preferences?
   1. What are they?
   2. What do you see as their strengths and weaknesses?

Conclusion

1. So one final question from me – is there anything else that you’d like to say on this topic – for example, are there things that you wish you’d added to your earlier responses, or is there anything that you thought we might cover but didn’t?

Thank you very much for your time today. If you’ve indicated you’re willing to be contacted again, we may be in touch in due course with an invitation to participate in a consensus- development exercise around improving planning for end-of-life care, and with findings from the study.

End-of-life care planning

Topic guide (group ii – people planning for their own end-of-life care)

Version 1.0

Introductory questions

We’re interested in talking to you about your knowledge and experience of planning for care and treatments at the end of life, and how people in the health and social care system support this process and ensure that people’s plans are acted on. I know this isn’t the easiest thing to talk about, so if there are any questions you don’t want to answer, that’s absolutely fine. If you need a break, just let me know. And if you want to stop the interview altogether, that’s no problem at all – you don’t need to give a reason and it won’t affect the care you receive from the NHS or any other provider.

1. Please could you tell me a little about yourself and your background, particularly as it relates to your experience of planning for end-of-life care.
2. What, if anything, do you know about the guidance from the National Institute for Health and Care Excellence (NICE) about end-of-life care provision?
   1. Specifically, what do you know about NICE’s guidance on supporting people in planning for care at the end of their lives?
   2. [Reassure if necessary that lack of knowledge about NICE guidance is not an issue for participation]
3. In general terms, what do you see as the key challenges in ensuring that people’s preferences about end-of-life care are understood and respected by the healthcare system?

Questions on key activities in planning for end-of-life care

In this study, we are particularly interested in the work involved in ensuring that people are able to make effective preparations for care and treatments at the end of life. This means, for example, giving people the opportunity to discuss their preferences with care providers, ensuring that these preferences are properly recorded and shared, and making sure that they are accessed and used by health and social care staff when caring for those people. We’d like to ask you questions about the various stages involved in this process. We realise that you may not know about all of these stages: if there are some questions you don’t feel well placed to answer, that’s fine – just let me know and we’ll move on to the next area.

1. First of all, **identifying people who may be coming towards the end of their life** – for example, entering what is likely to be the final year of their life. Can you tell me what you know about
   1. how healthcare providers identify people who might be in their final year of life?
   2. How healthcare providers might ensure that individuals are assessed for their end- of-life care and treatment needs in a timely and consistent manner?
2. Do you have any direct experience of this process? [If yes, prompt:]
   1. What did the process look like for you?
   2. How did you find the process?
3. What do you see as the key challenges in identifying people who may be approaching end of life?
   1. What (if any) do you see as the issues with healthcare staff leading this process?
   2. What (if any) do you see as the issues with relying on people approaching end of life themselves, and their carers, to identify who is approaching end of life?
4. Are there any particular groups who you think may be less easy to identify?
   1. Which groups and why?
   2. Can you think of ways of addressing this?
5. What would you see as important in making the identification process work well, for people approaching end of life, carers and staff?
6. Second, I’d like to ask you about **how to go about having important conversations with people who may be approaching the end of their life** about their preferences and needs for their care and treatment. Can you tell me what you know about how healthcare providers try to do this?
7. Do you have any direct experience of this process? [If yes, prompt:]
   1. What did the process look like for you?
   2. How did you find the process?
   3. How well prepared did you feel after the process, in terms of your prognosis and what you needed to do next?
8. What are the important considerations in *initiating* conversations like this?
   1. [If they have experience:] What worked well for you and what didn’t?
   2. Who do you feel is best placed to initiate these conversations – for example, a particular kind of healthcare professional, or someone with a particular relationship with the person nearing end of life?
9. What do you think are the most important things that the person planning for end-of-life care should be asked about in a conversation like this? [Prompt as necessary regarding:
   1. shared understanding of the diagnosis and prognosis
   2. goals of care and treatment –
      1. health outcomes important to the individual
      2. whether to prioritise extending life or to prioritise comfort and quality of life
   3. circumstances under which hospital admission should or should not be considered
   4. cardiopulmonary resuscitation, and any other specific treatments which the patient would or would not wish to receive
   5. preferred location of care
   6. involvement of family and carers
   7. other considerations]
10. What do you see as the main challenges in having these conversations? What puts people off having them?
11. What would you see as important in making these conversations go well, for people approaching end of life, carers and staff? [Prompt additionally if they have experience:]
    1. Did you feel that an appropriate amount of information was communicated?
    2. Did you feel that these conversations were held at the right time?
    3. Did you feel that you had time and space to discuss your care or treatment plan in the way you wanted?
12. Have you used any tools or resources for planning end-of-life care and treatments?
    1. What are they?
    2. What do you see as their strengths and weaknesses?
13. Next, I have some questions specifically about **advance care planning** – that is, how people would like to be cared for specifically in the event that they are no longer able to communicate their preferences. Can you tell me what you know about this process?
14. Do you have any direct experience of advance care planning? [If yes, prompt:]
    1. What did the process look like for you?
    2. How did you find the process?
    3. Did you feel that the advance care plan reflected your preferences accurately?
15. What, to your knowledge, are the main challenges in doing advance care planning well?
16. Have you used any tools or resources in advance care planning?
    1. What are they?
    2. What do you see as their strengths and weaknesses?
17. Last in terms of key activities in planning for end-of-life treatment and care, I’d like to ask you about **ensuring that people’s preferences are shared with and used** by other parts of the system. Can you tell me what you know about this?
18. Do you have any direct experience of trying to ensure that different health and social care staff in different parts of the system are aware of your end-of-life care preferences? [If yes, prompt:]
    1. How well do you feel this works?
    2. Were there any particular challenges with ensuring that everyone relevant was aware of your preferences?
19. Do you feel confident that your preferences on your end-of-life care and treatment will be respected?
    1. In what circumstances and settings do you feel they are more likely to be respected?
20. Have you used any tools or resources to help other people share and make use of your end-of-life care preferences?
    1. What are they?
    2. What do you see as their strengths and weaknesses?

Supplementary questions on ReSPECT

Finally, we’d like to ask you a few questions about one particular tool that is commonly used in advance care planning, the Recommended Summary Plan for Emergency Care and Treatment or ReSPECT process. You may not know much about this process, and you don’t need to answer these questions.

1. Do you have any experience of the ReSPECT process?
2. [For those who have used it:] Please could you tell me about your experience of ReSPECT.
   1. When did the ReSPECT conversation happen?
   2. Who did you have the conversation with?
   3. Did you feel the time and place that the conversation took place were appropriate?
   4. Did you feel you had the opportunity to express your views in the course of the ReSPECT process? Did you feel listened to?
   5. Were you happy with the recommendations recorded?
   6. Did you feel that the information provided during the ReSPECT process was sufficient?
   7. What do you feel could be done to improve the ReSPECT process?
3. Do you have any thoughts about how best to engage and educate the public about the ReSPECT process?
4. Overall, do you feel that the ReSPECT process can help with end-of-life planning and care?
   1. [If so:] Why?
   2. [If not:] Why not?

Conclusion

1. So one final question from me – is there anything else that you’d like to say on this topic – for example, are there things that you wish you’d added to your earlier responses, or is there anything that you thought we might cover but didn’t?

Thank you very much for your time today. If you’ve indicated you’re willing to be contacted again, we may be in touch in due course with an invitation to participate in a consensus- development exercise around improving planning for end-of-life care, and with findings from the study.

End-of-life care planning

Topic guide (group iii – people important to those planning for their own end- of-life care)

Version 1.0

Introductory questions

We’re interested in talking to you about your knowledge and experience of planning for care and treatments at the end of life for someone important to you, and how people in the health and social care system support this process and ensure that people’s plans are acted on. I know this isn’t the easiest thing to talk about, so if there are any questions you don’t want to answer, that’s absolutely fine. If you need a break, just let me know. And if you want to stop the interview altogether, that’s no problem at all – you don’t need to give a reason and it won’t affect the care that the person important to you receives from the NHS or any other provider.

1. Please could you tell me a little about yourself and your background, particularly as it relates to your experience of planning for end-of-life care for someone important to you.
2. What, if anything, do you know about the guidance from the National Institute for Health and Care Excellence (NICE) about end-of-life care provision?
   1. Specifically, what do you know about NICE’s guidance on supporting people in planning for care at the end of their lives?
   2. [Reassure if necessary that lack of knowledge about NICE guidance is not an issue

for participation]

1. In general terms, what do you see as the key challenges in ensuring that people’s preferences about end-of-life care are understood and respected by the healthcare system?

Questions on key activities in planning for end-of-life care

In this study, we are particularly interested in the work involved in ensuring that people are able to make effective preparations for care and treatments at the end of life. This means, for example, giving people the opportunity to discuss their preferences with care providers, ensuring that these preferences are properly recorded and shared, and making sure that they are accessed and used by health and social care staff when caring for those people. We’d like to ask you questions about the various stages involved in this process. We realise that you may not know about all of these stages: if there are some questions you don’t feel well placed to answer, that’s fine – just let me know and we’ll move on to the next area.

1. First of all, **identifying people who may be coming towards the end of their life** – for example, entering what is likely to be the final year of their life. Can you tell me what you know about
   1. how healthcare providers identify people who might be in their final year of life?
   2. How healthcare providers might ensure that individuals are assessed for their end- of-life care and treatment needs in a timely and consistent manner?
2. Do you have any direct experience of this process? [If yes, prompt:]
   1. What did the process look like for you and for the person important to you?
   2. How did you find the process?
3. What do you see as the key challenges in identifying people who may be approaching end of life?
   1. What (if any) do you see as the issues with healthcare staff leading this process?
   2. What (if any) do you see as the issues with relying on the people approaching end of life themselves, and their carers, to identify who is approaching end of life?
4. Are there any particular groups who you think may be less easy to identify?
   1. Which groups and why?
   2. Can you think of ways of addressing this?
5. What would you see as important in making the identification process work well, for people approaching end of life, carers and staff?
6. Second, I’d like to ask you about **how to go about having important conversations with people who may be approaching the end of their life** about their preferences and needs for their care and treatment. Can you tell me what you know about how healthcare providers try to do this?
7. Do you have any direct experience of this process? [If yes, prompt:]
   1. What did the process look like for you and the person important to you?
   2. How did you find the process?
   3. How well prepared did you feel after the process, in terms of the prognosis of the person important to you and what you needed to do next?
8. What are the important considerations in *initiating* conversations like this?
   1. [If they have experience:] What worked well for you and what didn’t?
   2. Who do you feel is best placed to initiate these conversations – for example, a particular kind of healthcare professional, or someone with a particular relationship with the person nearing end of life?
9. What do you think are the most important things that the person planning for end-of-life care should be asked about in a conversation like this? [Prompt as necessary regarding:
   1. shared understanding of the diagnosis and prognosis
   2. goals of care and treatment –
      1. health outcomes important to the individual
      2. whether to prioritise extending life or to prioritise comfort and quality of life
   3. circumstances under which hospital admission should or should not be considered
   4. cardiopulmonary resuscitation, and any other specific treatments which the patient would or would not wish to receive
   5. preferred location of care
   6. involvement of family and carers
   7. other considerations]
10. What do you see as the main challenges in having these conversations? What puts people off having them?
11. What would you see as important in making these conversations go well, for people approaching end of life, carers and staff? [Prompt additionally if they have experience:]
    1. Did you feel that an appropriate amount of information was communicated?
    2. Did you feel that these conversations were held at the right time?
    3. Did you feel that you and the person important to you had time and space to discuss their care or treatment plan in the way you both wanted?
12. Have you used any tools or resources for planning end-of-life care and treatments?
    1. What are they?
    2. What do you see as their strengths and weaknesses?
13. Next, I have some questions specifically about **advance care planning** – that is, how people would like to be cared for specifically in the event that they are no longer able to communicate their preferences. Can you tell me what you know about this process?
14. Do you have any direct experience of advance care planning? [If yes, prompt:]
    1. What did the process look like for you and the person important to you?
    2. How did you find the process?
    3. Did you feel that the advance care plan reflected the preferences of the person

important to you accurately?

1. What, to your knowledge, are the main challenges in doing advance care planning well?
2. Have you used any tools or resources in advance care planning?
   1. What are they?
   2. What do you see as their strengths and weaknesses?
3. Last in terms of key activities in planning for end-of-life treatment and care, I’d like to ask you about **ensuring that people’s preferences are shared with and used** by other parts of the system. Can you tell me what you know about this?
4. Do you have any direct experience of trying to ensure that different health and social care staff in different parts of the system are aware of the end-of-life care preferences of the person important to you? [If yes, prompt:]
   1. How well do you feel this works?
   2. Were there any particular challenges with ensuring that everyone relevant was aware of the preferences of the person important to you?
5. Do you feel confident that the preferences on end-of-life care and treatment of the person important to you will be respected?
   1. In what circumstances and settings do you feel they are more likely to be respected?
6. Have you used any tools or resources to help other people share and make use of the end-of-life care preferences of the person important to you?
   1. What are they?
   2. What do you see as their strengths and weaknesses?

Supplementary questions on ReSPECT

Finally, we’d like to ask you a few questions about one particular tool that is commonly used in advance care planning, the Recommended Summary Plan for Emergency Care and Treatment or ReSPECT process. You may not know much about this process, and you don’t need to answer these questions.

1. Do you have any experience of the ReSPECT process?
2. [For those who have used it:] Please could you tell me about your experience of ReSPECT.
   1. When did the ReSPECT conversation happen?
   2. Who did you have the conversation with?
   3. Did you feel the time and place that the conversation took place were appropriate?
   4. Did you feel that you and the person important to you had the opportunity to express your views in the course of the ReSPECT process? Did you feel listened to?
   5. Were you happy with the recommendations recorded?
   6. Did you feel that the information provided during the ReSPECT process was sufficient?
   7. What do you feel could be done to improve the ReSPECT process?
3. Do you have any thoughts about how best to engage and educate the public about the ReSPECT process?
4. Overall, do you feel that the ReSPECT process can help with end-of-life planning and care?
   1. [If so:] Why?
   2. [If not:] Why not?

Conclusion

1. So one final question from me – is there anything else that you’d like to say on this topic – for example, are there things that you wish you’d added to your earlier responses, or is there anything that you thought we might cover but didn’t?

Thank you very much for your time today. If you’ve indicated you’re willing to be contacted again, we may be in touch in due course with an invitation to participate in a consensus- development exercise around improving planning for end-of-life care, and with findings from the study.

End-of-life care planning

Topic guide (groups iv and v

– health and social care staff)

Version 1.0

Introductory questions

We’re interested in talking to you about your knowledge and experience of planning for care and treatments at the end of life, and how people in the health and social care system support this process and ensure that people’s plans are acted on. I know this isn’t the easiest thing to talk about, so if there are any questions you don’t want to answer, that’s absolutely fine. If you need a break, just let me know. And if you want to stop the interview altogether, that’s no problem at all – you don’t need to give a reason and it won’t affect your employment.

1. Please could you tell me a little about yourself and your background, particularly as it relates to your interest in and knowledge about end-of-life care.
2. What, if anything, do you know about the guidance from the National Institute for Health and Care Excellence (NICE) about end-of-life care provision?
   1. Specifically, what do you know about NICE’s guidance on supporting people in planning for care at the end of their lives?
   2. [Reassure if necessary that lack of knowledge about NICE guidance is not an issue for participation]
3. In general terms, what do you see as the key challenges in ensuring that people’s preferences about end-of-life care are understood and respected by the healthcare system?

Questions on key activities in planning for end-of-life care

In this study, we are particularly interested in the work involved in ensuring that people are able to make effective preparations for care and treatments at the end of life. This means, for example, giving people the opportunity to discuss their preferences with care providers, ensuring that these preferences are properly recorded and shared, and making sure that they are accessed and used by health and social care staff when caring for those people. We’d like to ask you questions about the various stages involved in this process. We realise that you may not know about all of these stages: if there are some questions you don’t feel well placed to answer, that’s fine – just let me know and we’ll move on to the next area.

1. First of all, **identifying people who may be coming towards the end of their life** – for example, entering what is likely to be the final year of their life. Can you tell me what you know about
   1. how healthcare providers identify people who might be in their final year of life?
   2. How healthcare providers might ensure that individuals are assessed for their end- of-life care and treatment needs in a timely and consistent manner?
2. Do you have any direct experience of this process? [If yes, prompt:]
   1. What did the process look like for you as a health or social care professional?
   2. How did you find the process?
3. What do you see as the key challenges in identifying people who may be approaching end of life?
   1. What (if any) do you see as the issues with systematically screening patient notes or data to identify people approaching end of life?
   2. What (if any) do you see as the issues with identifying people opportunistically when they come into contact with the health service (for example appointments with GPs or attendance at the ED)?
   3. What (if any) do you see as the issues with relying on patients and carers to identify who is approaching end of life?
4. Are there any particular groups who you think may be less easy to identify?
   1. Which groups and why?
   2. Can you think of ways of addressing this?
5. What would you see as important in making the identification process work well, for patients, carers and staff?
6. Have you used any tools or resources for this process?
   1. What are they?
   2. What do you see as their strengths and weaknesses?
7. Second, I’d like to ask you about **how to go about having important conversations with people who may be approaching the end of their life** about their preferences and needs for their care and treatment. Can you tell me what you know about how healthcare providers try to do this?
8. Do you have any direct experience of this process? [If yes, prompt:]
   1. What did the process look like for you as a health or social care professional?
   2. How did you find the process?
9. What are the important considerations in *initiating* conversations like this?
   1. [If they have experience:] What worked/has worked well for you in the past and what didn’t?
   2. Who do you feel is best placed to initiate these conversations – for example, a particular kind of healthcare professional, or someone with a particular relationship with the person nearing end of life?
10. What do you think are the most important things that the patient should be asked about in a conversation like this? [Prompt as necessary regarding:
    1. shared understanding of the diagnosis and prognosis
    2. goals of care and treatment –
       1. health outcomes important to the individual
       2. whether to prioritise extending life or to prioritise comfort and quality of life
    3. circumstances under which hospital admission should or should not be considered
    4. cardiopulmonary resuscitation, and any other specific treatments which the patient would or would not wish to receive
    5. preferred location of care
    6. involvement of family and carers
    7. other considerations]
11. What do you see as the main challenges in having these conversations? What do you think puts patients off having them? What do you think puts clinicians off having them?
12. What would you see as important in making these conversations go well, for patients, carers and staff? [Prompt additionally if they have experience:]
    1. Did you feel that an appropriate amount of information was communicated?
    2. Did you feel that these conversations were held at the right time?
    3. Did you feel that the person approaching end of life had time and space to discuss their care or treatment plan in the way they wanted?
13. Have you used any tools or resources for this process?
    1. What are they?
    2. What do you see as their strengths and weaknesses?
14. Next, I have some questions specifically about **advance care planning** – that is, how

people would like to be cared for specifically in the event that they are no longer able to communicate their preferences. Can you tell me what you know about this process?

1. Do you have any direct experience of advance care planning? [If yes, prompt:]
   1. What did the process look like for you as a health or social care professional?
   2. How did you find the process?
2. What are your views on how advance care plans should be documented?
3. What, to your knowledge, are the main challenges in doing advance care planning well?
4. Have you used any tools or resources in advance care planning?
   1. What are they?
   2. What do you see as their strengths and weaknesses?
5. Last in terms of key activities in planning for end-of-life treatment and care, I’d like to ask you about **ensuring that people’s preferences are shared with and used** by other practitioners. Can you tell me what you know about this?
6. Do you have any direct experience of this process? [If yes, prompt:]
   1. Was this as someone sharing an individual’s preferences or as someone trying to use existing plans?
   2. What did the process look like?
   3. How well did you feel it worked?
7. What do you see as the main challenges in:
   1. documenting people’s preferences appropriately?
   2. ensuring they are shared with the right people and organisations?
   3. ensuring they are acted upon at the right time in people’s end-of-life care?
   4. ensuring they result in access to the right services, for example hospice and palliative care?
8. What tends to help in:
   1. documenting people’s preferences appropriately?
   2. ensuring they are shared with the right people and organisations?
   3. ensuring they are acted upon at the right time in people’s end-of-life care?
   4. ensuring they result in access to the right services, for example hospice and palliative care?
9. In your experience, what steps are in place to ensure that copies of advance care plans are kept at a person’s place of residence and are accessible upon admission to a hospital, care home or hospice?
10. Have you used any tools or resources to help people share and make use of end-of-life care preferences?
    1. What are they?
    2. What do you see as their strengths and weaknesses?

Supplementary questions on ReSPECT

Finally, we’d like to ask you a few questions about one particular tool that is commonly used in advance care planning, the Recommended Summary Plan for Emergency Care and Treatment or ReSPECT process. You may not know much about this process, and you don’t need to answer these questions.

1. Do you have any experience of the ReSPECT process? [If no, proceed to Conclusion section]
2. How much do you think using the ReSPECT process helps identify patients who would benefit from planning about care and treatments towards the end of life? Why?
3. How much do you think the ReSPECT process helps you to conduct conversations with patients about care and treatment towards the end of life? Why?
4. How much do you think the ReSPECT process helps you document recommendations about care and treatment for patients towards the end of life? Why?
5. What do you think is good about the ReSPECT process, and what could be done to improve it?
6. Do you have any thoughts about how best to engage and educate the public about the ReSPECT process?
7. Overall, do you feel that the ReSPECT process can help with end-of-life planning and care?
   1. [If so:] Why?
   2. [If not:] Why not?

Conclusion

1. So one final question from me – is there anything else that you’d like to say on this topic – for example, are there things that you wish you’d added to your earlier responses, or is there anything that you thought we might cover but didn’t?

Thank you very much for your time today. If you’ve indicated you’re willing to be contacted again, we may be in touch in due course with an invitation to participate in a consensus- development exercise around improving planning for end-of-life care, and with findings from the study.
